# ROS-Mediated Unfolded Protein Response Activation Drives Hepatocyte Apoptosis in Mesaconitine-Induced Liver Injury

**DOI:** 10.3390/toxics13030155

**Published:** 2025-02-23

**Authors:** Jin Tian, Yubin Zhuang, Yinuo Liu, Yihong Zheng, Xuyang Liu, Shiyu Lin, Chenhua Zheng, Zekai Wu

**Affiliations:** 1 Key Laboratory of Gastrointestinal Cancer, Ministry of Education, School of Basic Medical Sciences, Fujian Medical University, Fuzhou 350122, China; tianjin1128@fjmu.edu.cn (J.T.); liuyinuo@stu.fjmu.edu.cn (Y.L.); zhengyihong@stu.fjmu.edu.cn (Y.Z.); lxydsgyx@163.com (X.L.); linshiyudmvyx@163.com (S.L.); 2 Laboratory Animal Center, Fujian Medical University, Fuzhou 350122, China; zyb880@fjmu.edu.cn; 3 Experiment Teaching Center of Basic Medical Sciences, School of Basic Medical Sciences, Fujian Medical University, Fuzhou 350122, China

**Keywords:** mesaconitine (MA), hepatotoxicity, inflammation, oxidative stress, apoptosis, unfolded protein response (UPR)

## Abstract

Mesaconitine (MA), a predominant diterpenoid alkaloid isolated from Aconitum species, exhibits notable pharmacological properties but is simultaneously associated with significant toxicological concerns, with its hepatotoxic mechanisms remaining largely unelucidated. In this study, zebrafish embryos were systematically exposed to MA to investigate its effects on hepatic development and function. Comprehensive analyses of liver morphology, inflammatory response, oxidative stress, and apoptotic pathways were conducted. MA induced dose-dependent hepatotoxicity, manifesting in a significant reduction in liver size and a marked downregulation of liver-specific genes, including *tfa*, *cp*, *hhex*, and *fabp10a*. The presence of oxidative stress was substantiated by elevated reactive oxygen species (ROS) levels, while hepatic inflammation was characterized by enhanced neutrophil infiltration and the upregulation of proinflammatory mediators, particularly *il1b* and *tnfa*. A global transcriptome analysis revealed the substantial upregulation of unfolded protein response (UPR)-associated genes, notably *hsp90b1*, *hspa5*, and *hspb9*, indicating that MA-induced oxidative stress triggered endoplasmic reticulum (ER) stress and subsequent UPR activation. Prolonged ER stress ultimately resulted in hepatocyte apoptosis, as demonstrated by the increased expression of the pro-apoptotic genes *casp3a*, *casp3b*, and *baxa*. These findings elucidate the molecular mechanisms underlying MA-induced hepatotoxicity and identify potential therapeutic targets for preventing and treating liver injury associated with Aconitum alkaloid exposure.

## 1. Introduction

Traditional Chinese medicines derived from Aconitum species, such as *Aconitum carmichaelii Debx.* (Fuzi), exhibit analgesic, anti-inflammatory, antidepressant, and vasodilatory properties [1,2,3]. However, natural products extracted from Aconitum plants, particularly Aconitum alkaloids, possess potent toxic effects. Current research on the toxicity of Aconitum alkaloids primarily focuses on their impact on the cardiovascular and central nervous systems, with limited studies addressing hepatotoxicity mechanisms. Preclinical studies have demonstrated clear dose-dependent hepatotoxicity associated with Aconitum alkaloids [4]. Moreover, several investigations into the toxicity resulting from a single or prolonged oral administration of Aconitum extract in rodents have reported instances of liver injury [5,6]. Consequently, it is imperative to conduct a systematic study of the hepatotoxicity mechanisms of Aconitum alkaloids to enable their safe clinical application.

MA is one of the principal diterpenoid alkaloids found in Aconitum species, exhibiting significant pharmacological effects while also being highly toxic. MA can penetrate the blood–brain barrier through proton-coupled organic cation antiporter mechanisms, leading to neurotoxicity [7]. Studies have demonstrated that MA induces neurotoxicity in zebrafish and HT22 cells, resulting in oxidative stress and mitochondrial dysfunction [8]. Additionally, MA significantly reduces the survival rate of H9C2 rat cardiomyocytes by inducing calcium influx, which subsequently decreases the mitochondrial membrane potential and causes nuclear damage [9]. Following MA exposure, cardiomyocytes from SD rats exhibit marked coagulative necrosis, with abnormal levels of polyunsaturated fatty acids (PUFAs) and altered expression of peroxisome proliferator-activated receptor α (PPARα) pathway-related proteins [10]. MA can induce cardiotoxicity and apoptosis, affecting cardiovascular-related genes such as *tbx5*, *gata4*, and *nkx2.5* in embryonic zebrafish [11]. Furthermore, a post-mortem analysis of patients who succumbed to Aconitine alkaloid poisoning revealed higher concentrations of MA in the liver and kidneys, reaching 960.9 ng/g and 776.9 ng/g, respectively [12]. In rats, MA has been shown to cause hepatocyte necrosis and inflammatory cell infiltration [13]. A network toxicological analysis indicated that MA affects pathways such as HIF-1, MAPK, PI3K-Akt, and FoxO signaling by modulating targets including ALB, AKT1, CASP3, and IL2. These findings provide partial insight into MA’s hepatoxicity; however, the exact mechanisms of this, particularly in vivo, remain to be elucidated.

In this study, using zebrafish as a model organism, we uncovered MA’s hepatotoxic effects. Our findings demonstrated that exposure to varying concentrations of MA significantly impaired liver development in zebrafish embryos, as evidenced by reduced liver size, the increased infiltration of neutrophils into the liver, elevated levels of ROS, and enhanced hepatocyte apoptosis. To elucidate the underlying mechanisms of MA-induced hepatotoxicity, we performed transcriptome sequencing on control and MA-treated zebrafish embryos. By constructing a comprehensive molecular profile, we discovered a significant upregulation of genes associated with UPR, including *hsp90b1*, *hspa5*, and *hspb9*, in response to MA treatment. These findings suggested that MA induced oxidative stress and hepatocyte apoptosis through the activation of ER stress and the UPR signaling pathway. Collectively, this study not only revealed novel molecular mechanisms underlying MA-induced hepatotoxicity but also provided valuable insights into potential diagnostic and therapeutic strategies for liver dysfunction associated with MA exposure.

## 2. Materials and Methods

### 2.1. Zebrafish Husbandry

Wild-type zebrafish (AB strain); transgenic lines *Tg(mpx:EGFP)* which specifically label neutrophils; and *Tg(fabp10a:dsred)* which specifically label hepatocytes were obtained from the China Zebrafish Resource Center (CZRC) in Wuhan, China. The zebrafish were maintained at a temperature of 28 ± 0.5 °C under a light/dark cycle of 14 h of light and 10 h of dark. Water changes were performed twice daily to ensure optimal water quality, and the fish were fed freshly hatched brine shrimp twice per day. All procedures adhered to the guidelines established by the Animal Care and Use Committee of Fujian Medical University.

### 2.2. Chemical Exposure

To assess MA’s toxicity, healthy embryos at 3 dpf were transferred into six-well plates, with a density of 40 embryos per well, and subsequently treated with MA (Must Bio-Technology, Chengdu, China). The exposure concentrations of MA utilized in this study were 0 μM, 2.5 μM, 5 μM, and 7.5 μM.

### 2.3. Toxicity Analysis of MA

The number of dead and deformed zebrafish embryos was recorded at 24 h post treatment (24 hpt) and 48 hpt, from which the survival rate and malformation rate were calculated. Heart rate was measured at 48 hpt. Using a microscope for observation, we manually counted the number of heartbeats over a 1 min period. Images were captured using a fluorescence stereomicroscope (Nikon, Shinagawa-ku, Japan), following anesthesia with a 0.02% tricaine solution. Liver size was assessed using *Tg(fabp10a:dsred)* transgenic zebrafish larvae at 48 hpt. Additionally, *Tg(mpx:EGFP)* transgenic zebrafish larvae at 48 hpt were selected for neutrophil counting within the liver region. The pericardial area and liver size were analyzed utilizing the ImageJ software (version number: 1.53t).

### 2.4. Oxidative Stress Detection

Zebrafish larvae at 48 hpt underwent treatment with the DCFH-DA fluorescent probe (Beyotime, Shanghai, China) and were incubated in the dark at 28.5 °C for 30 min before thorough washing with PBS. After standardizing exposure values across samples, fluorescence images were captured using a fluorescence stereomicroscope. The fluorescence intensity of the liver region for each group was quantified using the ImageJ software.

### 2.5. Quantitative Real-Time PCR (qRT-PCR)

Total RNA was extracted from zebrafish larvae employing TRIzol reagent (Invitrogen, CA, USA, Xiamen, China), following a previously reported protocol [14]. RNA was reverse-transcribed into cDNA utilizing a reverse transcription kit (Accurate Biology, Changsha, China). qRT-PCR analysis was conducted using SYBR Green Pro Taq HS Premix (Accurate Biology, Changsha, China), with cDNA serving as the template. The reactions were conducted on an Agilent AriaMX Real-Time PCR System (Agilent Santa Clara, CA, USA). The sequences of PCR primers are detailed in Table 1. The relative quantification analysis of target gene expression levels employed the 2^−ΔΔCt^ method [15].

### 2.6. Transcriptome Sequencing

Total RNA was extracted from zebrafish larvae in both the control group and 5 μM MA-treated group, followed by sequencing performed by Seqhealth Technology Co., Ltd. (Wuhan, China). Sequencing was conducted using the Novaseq6000 platform (Illumina, San Diego, CA, USA) with a read length of 150 base pairs. The DESeq2 software (version number: 1.28.1) package was employed to analyze differentially expressed genes (DEGs). Gene Ontology (GO) and Kyoto Encyclopedia of Genes and Genomes (KEGG) analyses were performed using the DAVID database. A heatmap was generated on https://www.bioinformatics.com.cn (last accessed on 10 December 2024).

### 2.7. Cell Apoptosis Detection

Acridine orange (AO) staining was utilized to identify apoptotic cells in zebrafish larvae. An AO dye solution at a concentration of 5 μg/mL was administered to each group and incubated at 28.5 °C for 20 min. Subsequently, the larvae were washed twice with PBS for three minutes each time. Zebrafish larvae were then photographed under a fluorescence stereomicroscope, and the fluorescence intensity within the liver region of each group was quantified using the ImageJ software.

### 2.8. Statistical Analysis

Statistical analyses were performed using GraphPad Prism 9.0. Group differences were assessed through one-way ANOVA for multiple group comparisons. Data are expressed as means ± standard deviation (SD). Statistical significance relative to the control group is denoted as *p* < 0.05 (*), *p* < 0.01 (**) or *p* < 0.001 (***).

## 3. Results

### 3.1. Effects of MA on Zebrafish Embryos Development

**Figure 1:**
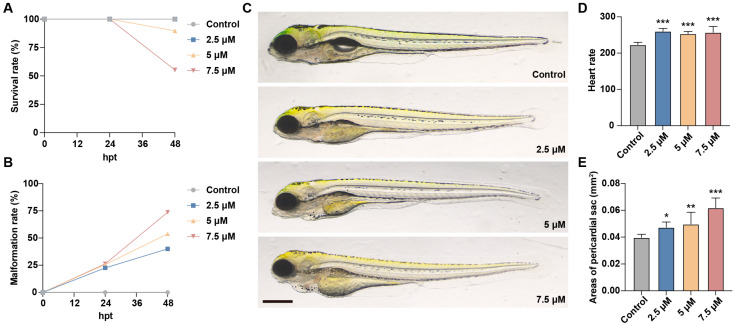
Effects of MA on zebrafish embryo survival and development. (**A**,**B**) Survival rate (**A**) and malformation rate (**B**) of zebrafish larvae exposed to 0 μM, 2.5 μM, 5 μM, and 7.5 μM of MA at 24 hpt and 48 hpt. (**C**) Representative images of control and MA-treated zebrafish larvae. Scale bar: 400 µm. (**D**,**E**) Statistical analysis of heart rate (**D**) and pericardial cavity area (**E**) of control and MA-treated zebrafish larvae at 48 hpt. One-way ANOVA–Dunnett test; * *p* < 0.05, ** *p* < 0.01, *** *p* < 0.001. Error bars represent standard deviation.

To investigate the effects of MA on zebrafish embryo survival and development, embryos at 3 dpf were exposed to MA at concentrations of 0 μM, 2.5 μM, 5 μM, and 7.5 μM for a duration of 48 h. The survival rates and morphological abnormalities were observed and recorded at 24 hpt and 48 hpt. Additionally, the heart rate and pericardial cavity area were measured at 48 hpt to assess developmental impacts. The results showed that with the increase in treatment time and MA concentration, the embryos’ survival rate decreased, and their malformation rate significantly increased (Figure 1A,B). MA concentrations of less than 2.5 μM did not cause the zebrafish larvae to die. The survival rates at MA concentrations of 5 μM and 7.5 μM were 89.74% and 55.26%, respectively. Moreover, increasing the MA concentration resulted in pericardial edema and elevated heart rates, highlighting its cardiotoxic effects, which align with previous studies’ findings (Figure 1C–E).

### 3.2. MA Impaired Liver Development in Zebrafish Embryos

**Figure 2:**
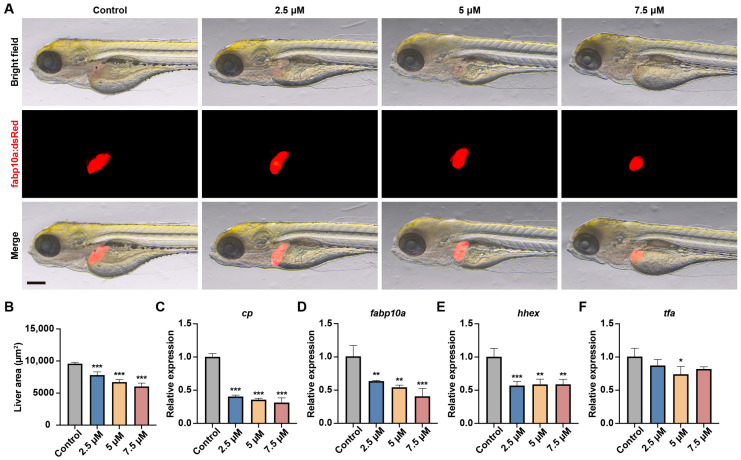
MA impaired liver development in zebrafish embryos. (**A**) Representative images of the Tg(*fabp10a:DsRed*) transgenic zebrafish larvae in the control and MA-treated groups at 48 hpt. Scale bar: 200 µm. (**B**) Statistical analysis of the liver area of zebrafish larvae. (**C**–**F**) qRT-PCR showing the effects of MA treatment on the expression of *cp* (**C**), *fabp10a* (**D**), *hhex* (**E**), and *tfa* (**F**) in zebrafish larvae. One-way ANOVA–Dunnett test; * *p* < 0.05, ** *p* < 0.01, *** *p* < 0.001. Error bars represent standard deviation.

To investigate MA’s effects on liver development, we utilized the Tg(fabp10:DsRed) transgenic zebrafish line to observe the liver morphology. Compared with the control group, the fluorescent area of the liver region in the MA-treated groups was significantly reduced (Figure 2A,B). We further analyzed liver development gene expression using qRT-PCR and found that genes related to liver development, such as tfa, cp, hhex, and fabp10a, were significantly downregulated (Figure 2C–F). These findings indicate that MA exerts toxic effects on liver development in a dose-dependent manner. 

### 3.3. MA Induced Liver Inflammation in Zebrafish Embryos

**Figure 3:**
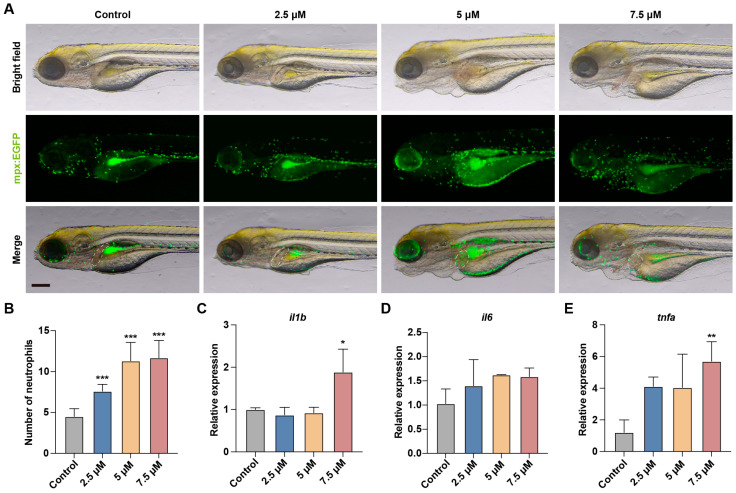
MA Induces liver inflammation in zebrafish embryos. (**A**) Representative images of the *Tg(mpx:EGFP)* transgenic zebrafish larvae in the control and MA-treated groups at 48 hpt. Scale bar: 200 µm. (**B**) Statistical analysis of the number of neutrophils in the liver area of zebrafish larvae. (**C**–**E**) qRT-PCR showing the effects of MA treatment on the expression of *il1b* (**C**), *il6* (**D**)*,* and *tnfa* (**E**) in zebrafish larvae. One-way ANOVA–Dunnett test, * *p* < 0.05, ** *p* < 0.01, *** *p* < 0.001. Error bars represent standard deviation. The white dashed line indicates the liver region.

Neutrophils play a crucial role in maintaining immune system function and responding to infections. After tissue damage, neutrophils rapidly migrate to the damaged area, releasing cytokines and ROS to trigger inflammatory responses [16]. To investigate whether MA induced inflammation in the liver region, we used the *Tg*(*mpx:EGFP*) transgenic zebrafish line to observe changes in the number of neutrophils in the liver area. As shown in Figure 3A,B, the neutrophil number significantly increased in the MA-treated groups compared to that in the control group, displaying a clear dose-dependent relationship. In addition, we further analyzed the expression of inflammatory mediators such as *il6*, *il1b*, and *tnfa* in the zebrafish embryos. The results revealed a significant upregulation of *il1b* and *tnfa* (Figure 3C–E). These findings indicate that MA treatment induces inflammation in the liver region of zebrafish larvae.

### 3.4. MA Induced Oxidative Stress Response in Zebrafish Embryos

**Figure 4:**
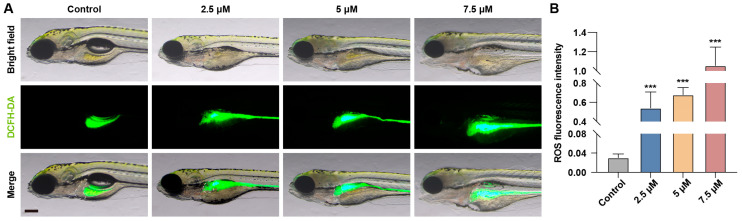
MA induces oxidative stress response. (**A**) Representative images of ROS staining results of control and MA-treated zebrafish larvae at 48 hpt using DCFH-DA probes. Scale bar: 200 µm. (**B**) Statistical analysis of fluorescence intensity in the liver area. One-way ANOVA–Dunnett test; *** *p* < 0.001. Error bars represent standard deviation. The white dashed line indicates the liver region.

Cells naturally produce ROS as byproducts of normal metabolism. However, when cells are exposed to internal or external stress, ROS production increases, disrupting the balance between oxidative and antioxidant systems and ultimately leading to oxidative stress [17]. To verify whether MA induces oxidative stress in hepatocytes, we used DCFH-DA probes to measure the ROS levels in the liver region. The results showed a significant increase in fluorescence intensity in the liver area of zebrafish embryos treated with MA (Figure 4A,B). This indicates that ROS levels were markedly elevated in the treatment groups, confirming that MA induces oxidative stress in hepatocytes.

### 3.5. MA Induced UPR in Zebrafish Embryos

**Figure 5:**
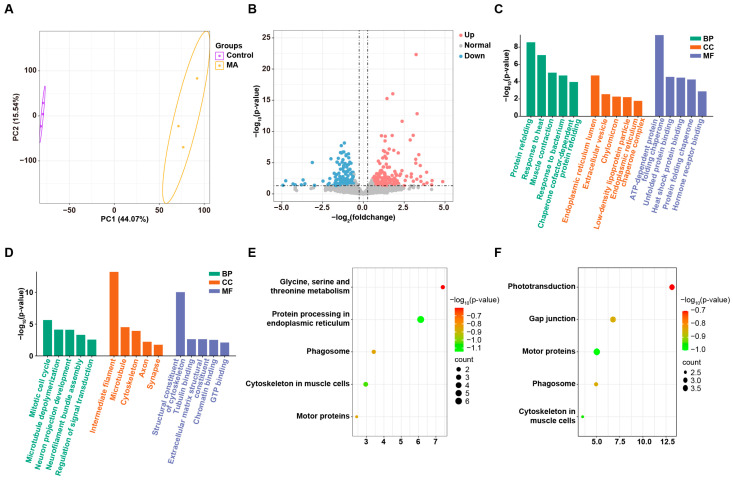
Molecular diversity of zebrafish embryos following MA treatment. (**A**) PCA analysis of RNA sequencing results showing differences in gene expression between control and MA-treated zebrafish embryos. (**B**) Volcano plot showing differentially expressed genes between control and MA-treated embryos. Significance was defined as a fold change greater than 1.5 or less than 0.67, with a *p*-value less than 0.05. (**C**,**D**) Representative GO terms of upregulated (**C**) and downregulated (**D**) genes after MA treatment. (**E**,**F**) Representative KEGG entries for upregulated (**E**) and downregulated (**F**) genes after MA treatment.

To investigate the mechanisms underlying MA-induced liver toxicity, we conducted transcriptome sequencing on zebrafish embryos at 5 dpf to analyze the DEGs between the control and MA-treated groups. A principal component analysis (PCA) of the sequencing data revealed a clear separation between the gene expression profiles of the control and MA-treated embryos (Figure 5A). We utilized 17,549 genes with non-zero variance as input variables for the PCA. Principal Component 1 (PC1) accounted for 44. 07% of the total variance, while PC2 explained 15. 54%. A total of 345 DEGs were identified in the MA-treated group, including 184 upregulated and 161 downregulated genes (Figure 5B), indicating significant alterations in liver gene expression following MA exposure. To further explore the functions of these DEGs, a GO analysis was performed, identifying associated biological processes (BPs), cellular components (CCs), and molecular functions (MFs). The results revealed that the upregulated genes were primarily associated with BPs such as protein refolding, heat response, muscle contraction, bacterial response, and chaperone cofactor-dependent protein refolding (Figure 5C). The CC terms were enriched in the endoplasmic reticulum lumen, extracellular vesicles, chylomicrons, low-density lipoprotein (LDL) particles, and endoplasmic reticulum chaperone complexes. The MF terms included ATP-dependent protein folding, unfolded protein binding, heat shock protein binding, protein folding chaperones, and hormone receptor binding. The downregulated genes were mainly involved in BPs such as the mitotic cell cycle, microtubule depolymerization, neuron projection development, neurofilament bundle assembly, and signal transduction regulation. The CCs were primarily located in the intermediate filaments, microtubules, cytoskeleton, axons, and synapses. The MFs mainly included structural components of the cytoskeleton, tubulin binding, extracellular matrix structural constituent, chromatin binding, and GTP binding (Figure 5D). To identify the metabolic pathways in which these DEGs play critical roles, we performed a KEGG pathway enrichment analysis to further elucidate their biological functions. Following MA treatment, the protein processing pathway in the endoplasmic reticulum was significantly upregulated, consistent with the results of our GO enrichment analysis (Figure 5E). Genes associated with phototransduction, gap junctions, and motor proteins were downregulated (Figure 5F). Collectively, these findings suggested that MA activated UPR in the zebrafish embryos.

**Figure 6:**
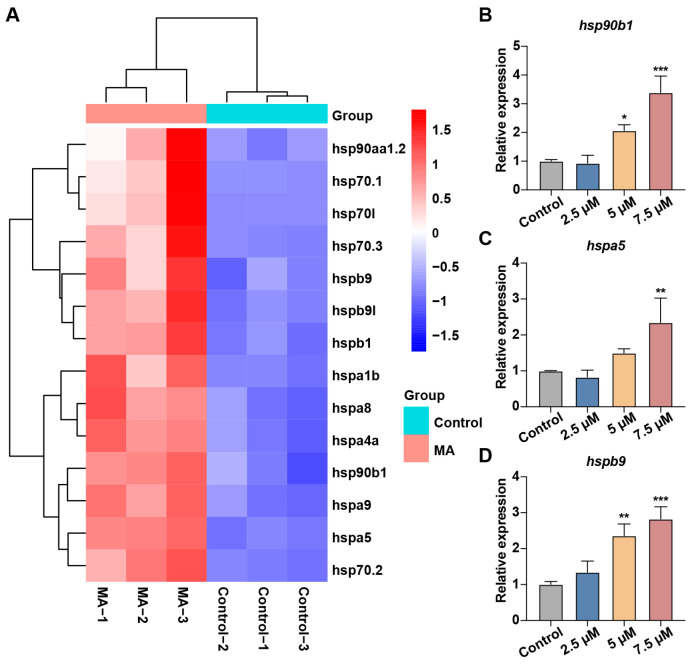
MA induced UPR in zebrafish embryos. (**A**) Heatmap showing scaled expression levels of hsp family genes in the control and MA-treated groups. (**B**–**D**) qRT-PCR analysis showing increased expression levels of *hsp90b1* (**B**), *hspa5* (**C**), and *hspb9* (**D**) in zebrafish larvae treated with MA compared to the control group. One-way ANOVA–Dunnett test; * *p* < 0.05, ** *p* < 0.01, *** *p* < 0.001. Error bars represent standard deviation.

To investigate the mechanism by which oxidative stress induces UPR, we analyzed the changes in HSP expression between the control and MA-treated groups. The heatmap showed that HSP gene expression was upregulated in the MA-treated group (Figure 6A). We further validated these findings using qRT-PCR, which confirmed the significant increase in *hsp90b1*, *hspa5*, and *hspb9* expression levels (Figure 6B–D).

### 3.6. MA Induces Hepatocytes Apoptosis in Zebrafish Embryos

**Figure 7:**
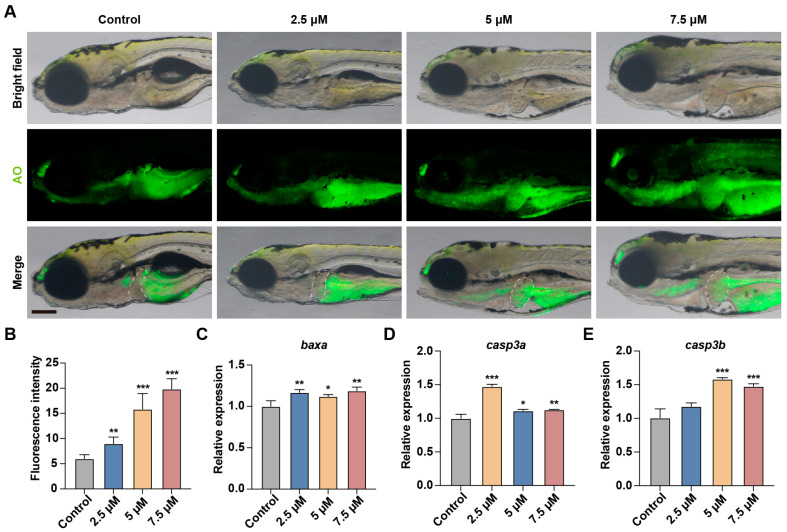
MA induces hepatocyte apoptosis in zebrafish embryos. (**A**) Representative images of AO staining results in control and MA-treated zebrafish larvae at 48 hpt. Scale bar: 200 µm. (**B**) Statistical analysis of fluorescence intensity in the liver area. (**C**–**E**) qRT-PCR showing the effects of MA treatment on the expression of *baxa* (**C**), *casp3a* (**D**), and *casp3b* (**E**) in zebrafish larvae. One-way ANOVA–Dunnett test; * *p* < 0.05, ** *p* < 0.01, *** *p* < 0.001. Error bars represent standard deviation. The white dashed line indicates the liver region.

Persistent ER stress, which fails to restore ER homeostasis, activates pro-apoptotic pathways and ultimately leads to apoptosis [18]. To evaluate hepatocyte apoptosis, we employed acridine orange, a cell-permeable nucleic acid-binding fluorochrome that exhibits green fluorescence upon binding to DNA in apoptotic cells when visualized under fluorescence microscopy. A quantitative analysis revealed that MA exposure induced a significant dose-dependent increase in the fluorescence intensity within the hepatic region compared to the control group, indicating substantial hepatocyte apoptosis (Figure 7A,B). Furthermore, the molecular analysis demonstrated the significant upregulation of key pro-apoptotic genes, including *casp3a*, *casp3b*, and *baxa* (Figure 7C–E). These findings collectively demonstrate that MA exposure induces dose-dependent hepatotoxicity characterized by enhanced hepatocyte apoptosis.

## 4. Discussion

In this study, we used zebrafish as a model organism to explore MA’s toxic effects on the liver. Our findings demonstrated that exposure to varying MA concentrations significantly impaired liver development in zebrafish embryos, as evidenced by the reduced liver size, increased infiltration of neutrophils into the liver, elevated ROS levels, and enhanced hepatocyte apoptosis. Moreover, we observed a significant upregulation of UPR-associated genes, including *hsp90b1*, *hspa5*, and *hspb9*, in response to MA treatment. These findings suggest that MA induced oxidative stress and hepatocyte apoptosis through the activation of endoplasmic reticulum stress and the UPR signaling pathway.

We initially investigated the developmental toxicity of MA exposure in zebrafish embryos. Both the mortality and malformation rates showed a positive correlation with the MA concentration. The pericardial area expansion correlated with increasing MA concentrations. While the heart rate showed a slight elevation, the changes were not markedly concentration-dependent. The increased heart rate may reflect enhanced energy metabolic demands, suggesting that MA accelerates zebrafish metabolism. These findings indicate that MA induces organ toxicity in zebrafish through both cardiotoxic and metabolic pathways, which aligns with previously reported observations of MA-induced cardiotoxicity.

To visually assess the MA-induced hepatotoxicity in vivo, we examined the liver size changes in *Tg(fabp10a:dsRed)* transgenic zebrafish, which specifically labels hepatocytes. At 5 dpf, we observed that the liver fluorescence area decreased with an increase in the MA concentration. Previous studies have shown that *tfa* is involved in tissue factor synthesis in the liver [19]; *cp* encodes ceruloplasmin, a glycoprotein primarily synthesized in the liver that transports copper ions and iron oxides, participates in iron metabolism and oxidative stress regulation, and maintains hepatocyte survival and function through oxidative stress repair [20]. *hhex* is crucial for liver specification, liver bud growth, and cell differentiation [21], while *fabp10a* encodes a fatty acid-binding protein (FABP) family member essential for hepatic fatty acid metabolism [22]. To further confirm that MA hindered liver development and metabolism, resulting in liver toxicity, we selected four genes, *tfa*, *cp*, *hhex,* and *fabp10a,* for qPCR detection. The results revealed the downregulation of all four genes, indicating suppressed liver development and metabolism. These findings suggest that one of MA’s hepatotoxic mechanisms involves the inhibition of genes essential for liver development and metabolism, resulting in reduced liver growth and impaired hepatic metabolic function.

In this study, we quantified the neutrophil numbers in the hepatic region to test whether MA induces hepatocellular damage and triggers an inflammatory response [23]. The statistical data showed that neutrophils significantly accumulated in the zebrafish liver area, with their numbers increasing in a concentration-dependent manner with MA treatment, supporting our previous findings regarding MA-induced hepatic injury. To further investigate the mechanism of the MA-induced inflammatory response at the transcriptional level, we examined the expression of inflammatory genes *il1b*, *tnfa*, and *il6* using qRT-PCR. The results revealed the significant upregulation of both *il1b* and *tnfa*, while *il6* showed an increasing trend without reaching statistical significance. Previous studies have established that *il1b* encodes the proinflammatory cytokine IL-1β, which regulates immune and inflammatory responses [24]. *tnfa* encodes TNF-α, a proinflammatory cytokine involved in immune regulation, inflammatory response, apoptosis, and anti-tumor processes [25]. Similarly, *il6* encodes IL-6, which enhances neutrophil function and immune cell activity while promoting and regulating the secretion of cytokines, adhesion molecules, and inflammatory mediators such as nitric oxide [26]. These findings indicate that MA induces inflammatory gene expression, promotes neutrophil recruitment to injury sites, and thereby activates the inflammatory response.

ROS are highly reactive oxygen-containing molecules that play crucial roles in cell signaling and physiological processes. Certain toxins can trigger excessive ROS generation, leading to oxidative stress, cellular damage, and cytotoxicity [27]. To investigate the MA-induced hepatotoxicity, we measured the ROS levels in zebrafish exposed to various MA concentrations using the DCFH-DA fluorescent probe. The results demonstrated a significant positive correlation between the fluorescence intensity in the liver region and the MA concentration, indicating that MA stimulates ROS production and disrupts the oxidation–antioxidation balance. This oxidative stress leads to hepatocellular damage and dysfunction, which may contribute to the observed reduction in the zebrafish liver size.

To further investigate MA’s hepatotoxicity, we performed transcriptome sequencing of the control and MA-treated zebrafish and identified the increased expression of several HSP family genes, including *hsp90b1*, *hspa5*, and *hspb9*. The HSP gene family encodes heat shock proteins that protect cells from stress-induced damage and function as molecular chaperones in protein folding, assembly, transport, and degradation. These proteins prevent protein misfolding and aggregation [28], playing crucial roles in the UPR [29]. The UPR is a cellular stress response mechanism activated by the accumulation of unfolded or misfolded proteins in the endoplasmic reticulum. The upregulation of HSP genes and subsequent increase in heat shock protein levels indicate that zebrafish activate anti-damage and UPR mechanisms to counter MA-induced cellular damage and protein synthesis disruption. To validate these findings, we performed a qRT-PCR analysis of the aforementioned genes. The results confirmed the upregulation of multiple HSP family genes following the MA treatment, suggesting that MA exposure causes protein and cellular damage while triggering the UPR. These findings indicate that MA interferes with normal protein folding in the hepatocyte endoplasmic reticulum, leading to misfolded protein accumulation and subsequent hepatotoxicity.

The ROS-triggered UPR can induce apoptosis [29,30], potentially contributing to zebrafish liver shrinkage. Using AO staining to detect cell apoptosis, we observed that the fluorescence intensity increased in a concentration-dependent manner with MA treatment, indicating MA-induced hepatocyte apoptosis. We focused on *casp3a*, *casp3b*, and *baxa*, which are apoptotic genes with broad regulatory roles in programmed cell death, hypothesizing their involvement in MA-mediated hepatocyte apoptosis [31,32,33]. To validate this hypothesis, we performed a qRT-PCR analysis of these genes. The results demonstrated the upregulation of all three apoptotic genes, providing strong experimental support for our hypothesis. These findings indicate that MA promotes hepatocyte apoptosis through the upregulation of key apoptotic genes including *casp3a*, *casp3b*, and *baxa*, further demonstrating its hepatotoxic effects.

## 5. Conclusions

Our findings demonstrate that MA induces hepatotoxicity in zebrafish, as evidenced by a reduced liver size, increased neutrophil infiltration, elevated ROS level, upregulation of UPR, and enhanced hepatocyte apoptosis. This study expands our understanding of MA’s toxic profile and elucidates the underlying mechanisms of its hepatotoxicity. Given MA’s use as a pharmaceutical component, these findings highlight the importance of careful dosage control to minimize potential adverse effects and underscore the need for appropriate risk management strategies in its clinical application.

## Figures and Tables

**Table 1:** Primer sequences of qRT-PCR in zebrafish.

Designed qRT-PCR Primer Sequences (5′ to 3′)			
	Gene	Forward	Reverse
Reference gene	*actb2*	CCCAAACCCAAGTTCAGCCA	ACCCACGATGGATGGGAAGA
Apoptosis-related genes	*baxa*	TGGCAAGTTCAACTGGG-GAA	ATAACTGCGGATTCCGTCCC
	*casp3a*	CCCAGTGGAGGCAGATTTCC	AGCATTGAGAC-GATGCAGGG
	*casp3b*	ACAACACCAGAAGCAGGACTT	TTTGCATCGCTTTGTCTGGC
Liver Development-related genes	*cp*	CGCTTCTGGAACCGTCAGTC	CTCGTTGCCTGGGCTTTCTT
	*fabp10a*	CCACCATGGACGGCAAGAAG	GACTGTCAGCGTCTCCACCA
	*hhex*	AATCCTCCGTCCACCGGTAA	GGGTGAACTGATGCTCGTCC
	*tfa*	GACTGCAGCTGCTCACACAA	TCTGCCTCTCACTCTCTGGG
Unfolded protein response-related genes	*hspa5*	CAGATCTGGCCAAAATGCGG	ATGACGGAGTGATGCGGTTT
	*hspb9*	TCCTCAACCTTCTCCAGGCT	CCTGGGACTCAGCAGATGAC
	*hsp90b1*	TCTGTGCACTTTTGGCGTTC	TCGATTCACTTCTGCCTGGA
Inflammation-related genes	*il1b*	TCTGCTCAGCCTGTGTGTTT	GAGACCCGCTGATCTCCTTG
	*il6*	ACTCAGAGACGAGCAGTTTGA	GCGGTCTGAAGGTTTGAGGA
	*tnfa*	TCACGCTCCATAAGACCCAG	AAATGGATGGCAGCCTTGGA

## Data Availability

The RNA-seq data are available on the NCBI Sequence Read Archive (SRA) database: PRJNA1202211. Other materials used in this study are available from the corresponding authors on reasonable request.

## Abbreviations

The following abbreviations are used in this manuscript:

MA	Mesaconitine
PUFAs	Polyunsaturated fatty acids
PPARα	Peroxisome proliferator-activated receptor α
ROS	Reactive oxygen species
UPR	Unfolded protein response
CZRC	China Zebrafish Resource Center
DEGs	Differentially expressed genes
GO	Gene Ontology
KEGG	Kyoto Encyclopedia of Genes and Genomes
AO	Acridine orange
SD	Standard deviation
PCA	Principal component analysis
BP	Biological processes
CC	Cellular components
MF	Molecular functions
LDL	Low-density lipoprotein
ER	Endoplasmic reticulum
HSP	Heat shock protein
FABP	Fatty acid binding protein
